# The impact of theory-based educational interventions: A three-arm randomized controlled trial comparing face-to-face, mobile-based, and control approaches on pap smear acceptance

**DOI:** 10.1016/j.pmedr.2025.103198

**Published:** 2025-08-09

**Authors:** Azar Bahrami, Mitra Rahimzadeh, Ali Safari-Moradabadi

**Affiliations:** a Student Research Committee, School of Health, Alborz University of Medical Sciences, Karaj, Iran; b Department of Epidemiology, School of Health, Alborz University of Medical Sciences, Karaj, Iran; c Department of Health Promotion and Education, School of Health, Alborz University of Medical Sciences, Karaj, Iran

**Keywords:** Pap smear, Screening, Education, Intervention, Cervical cancer, Mobile health

## Abstract

**Objective:**

This study aimed to evaluate the effectiveness of face-to-face and mobile-based educational interventions, grounded in the Health Belief Model (HBM), in promoting Pap smear acceptance.

**Methods:**

A three-arm randomized controlled trial was conducted among 135 women attending urban health centers under the supervision of the Zanjan Health Center in Alborz, Iran. Participants were randomly assigned to one of three groups: face-to-face education, mobile-based education via Telegram, and a control group receiving no immediate intervention. Statistical analyses included repeated measures ANOVA (RMANOVA) and analysis of covariance (ANCOVA) to adjust for confounding factors. The data collection period spanned from November 1, 2023, to September 5, 2024.

**Results:**

Both face-to-face and mobile-based interventions significantly improved awareness and HBM constructs compared to the control group. The mobile-based group demonstrated the greatest increase in perceived susceptibility and self-efficacy immediately post-intervention. Behavioral intention and actual Pap smear uptake also showed significant improvement in the intervention groups. However, perceived barriers did not differ significantly between the groups.

**Conclusions:**

Theory-based educational interventions—especially mobile-based strategies—effectively promote Pap smear acceptance. Their accessibility supports national screening efforts and aligns with global policies aimed at reducing disparities. Integrating mobile education into public health programs may enhance coverage and improve preventive outcomes.

## Highlights

• Mobile-based and face-to-face education improved Pap smear screening uptake.
• The Health Belief Model guided the intervention design for cervical cancer screening.
• Mobile-based education effectively enhanced awareness and self-efficacy.
• Behavioral intention and Pap smear uptake increased in intervention groups.
• Mobile interventions offer a scalable approach to cervical cancer prevention.

## 1 Introduction

Cancer is recognized as one of the most common and serious causes of death worldwide, with its incidence and prevalence continuously increasing (Massobrio et al., 2024). According to projections from the World Health Organization (WHO), cancer is expected to become the leading cause of death globally by 2030. A report released by GLOBOCAN in 2020 estimates that cancer cases will rise to approximately 28.4 million by 2040 (Balou et al., 2022). Specifically, cervical cancer is considered the third most common cancer among women and the fourth leading cause of cancer-related death in women worldwide (Hoshmndi et al., 2020). Cervical cancer is also a significant health concern in Iran, ranking among the most common cancers in women. Although its incidence is lower compared to many other countries, the associated mortality rate remains notable. A review indicated that the age-standardized incidence rate (ASR) of cervical cancer in Iran was approximately 2.4 cases per 100,000 women in 2016, with a marked increase in registered cases over the years (Eftekharzadeh et al., 2020). Furthermore, one study reported that cervical cancer accounted for approximately 3.46 % of deaths among women aged 15–59, underscoring its impact on female mortality (AziziKia et al., 2023). Despite the relatively low incidence, the age-standardized mortality rate (ASMR) for cervical cancer was reported at 1.5 deaths per 100,000 women, indicating a significant disease burden (Rostami and Nahvijou, 2022). Cervical cancer is highly preventable due to its extended preclinical stage, which allows for early detection and timely intervention (Nejati et al., 2020). Screening methods like the Pap smear can identify cervical lesions at an early stage, thus preventing progression to cancer (Sharifi et al., 2018). When diagnosed and treated during the precancerous stage, the survival rate can reach nearly 100 % (Zareai, 2014). However, screening rates remain low in most developing countries, with only about 5 % of women undergoing Pap smear testing, and less than 20 % in Iran (Ahadinezhad et al., 2024), compared to over 90 % in the United States.

**Fig. 1:**
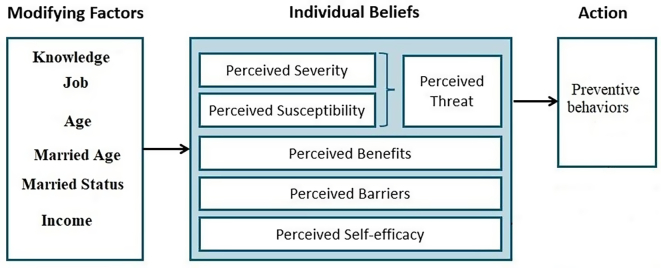
Theoretical Framework of Health Belief Model.

Women face various barriers in different societies—cultural, emotional, social, economic, and geographical—that hinder access to available screening services. These barriers range from insufficient financial resources to low health literacy and a lack of understanding of the importance of screening tests (Sharifi et al., 2018). Numerous researchers have identified low awareness as one of the primary reasons for the underutilization of Pap smear testing (Nazari et al., 2019). In such contexts, health education is an effective approach to increasing awareness and promoting health-enhancing behaviors (Shokouhi et al., 2021). It plays a critical role in encouraging women's participation in cervical cancer screening programs. However, to maximize the effectiveness of these interventions, they should be systematically designed using theoretical frameworks. One such widely applied framework is the Health Belief Model (HBM), which is commonly used to develop educational components for health interventions (Ghoreishi et al., 2019; Mokhtari Nouri et al., 2019) (Fig. 1). By targeting individual beliefs and perceptions, the model serves as a valuable tool for designing educational interventions that have been shown to increase awareness, shift health beliefs, and boost participation in cervical cancer screening and other preventive practices (Hatefnia and Ghazivakili, 2015).

This paper examines: (1) the theory's development and its role in explaining Pap smear behavior and early detection, and (2) the design of a three-arm randomized controlled trial (RCT) comparing face-to-face and mobile-based educational interventions with a control group to evaluate behavioral change.

## 2 Methods

### 2.1 Design and setting

This study employed a RCT with an open-label design, conducted across three parallel groups in urban health centers under the supervision of the Zanjan Health Center during 2023–2024. Ethical approval was obtained from Alborz University of Medical Sciences and Health Services (Ethics Code: IR.ABZUMS.REC.1402.294). The data collection period spanned from November 1, 2023, to September 5, 2024.

### 2.2 Participants

All urban health centers under the supervision of the Zanjan Health Center were eligible to participate in the study. Women were included if they met the following criteria: aged 21 years or older, married for at least three years, willing to participate in the study, psychologically healthy, literate, possessed a smartphone with internet access, and were members of virtual educational channels. Women were excluded if they died, relocated, expressed unwillingness to continue participation, lost internet or channel access, failed to complete the questionnaire, provided incomplete responses, or missed more than one in-person educational session. The same criteria applied to women with a history of cervical cancer, total hysterectomy, psychological disabilities, or those who had received similar training within the past year. The sample size was determined using G*Power software. Assuming an effect size of 0.15 for repeated measures analysis across three groups with three measurement points, a correlation of 0.5 between repeated measures, a 95 % confidence level, and 90 % statistical power, a total of 117 participants (39 per group) was required. To account for a potential 15 % attrition rate, the final sample size was increased to 135 participants, with 45 individuals randomly assigned to each of the three groups.

### 2.3 Randomization

At the start of the study, all urban health centers under the supervision of the Zanjan Health Center were identified based on the inclusion and exclusion criteria. These centers were geographically clustered to minimize contamination bias that could result from shared tracking and communication systems. Subsequently, one-stage randomization was performed by the study statistician in a random order within clusters, without stratification, at a 1:1:1 ratio. The clustersand therefore the health centers within each cluster, along with the participants registered at these centers—were then assigned to three equal groups.

### 2.4 Intervention and control procedures

Participants were randomly assigned to one of the three groups below:

**Group A (In-person):** This group received the intervention through in-person educational sessions incorporating lectures, discussions, and a question-and-answer format. Participants were divided into three subgroups of 15 individuals each. The sessions, each lasting 45 min, were held at urban health centers. A total of 12 sessions were conducted over four weeks, with each session focusing on a distinct topic.

**Group B (Mobile-based):** In this group, participants first received an explanation of the study's objectives, followed by obtaining oral or written consent. They were then be asked to join the virtual class using their active phone numbers. Education was delivered via the social media platform Telegram, as agreed upon with the participants. The program was implemented continuously and consistently by the researcher using a blended learning approach, which included online educational sessions featuring videos, animations, and PowerPoint presentations. An online Q& A was also conducted. To ensure participants were engaging with the educational content, an evaluation of the educational process was conducted two weeks after the intervention.

Although Telegram is officially restricted in Iran, it remained widely used by the public. To minimize selection bias, participants were included only if they had regular access to Telegram and were comfortable using it. This criterion was assessed during the initial screening process.

**Group C (Control group):** Participants in this group did not receive any instructional intervention during the study. Instead, they were provided with an educational package during a group session held at the conclusion of the study.

The educational program in this study employed an active approach based on a predefined lesson plan. Once participants were selected, they were contacted to arrange the timing and location of the sessions. The sessions covered topics including women's health, cervical cancer and its signs, the epidemiology of the disease, the benefits of Pap smear screening, and strategies for overcoming barriers to screening. The content also incorporated the “Must Know” instructional packages from the Iranian Ministry of Health, focusing on cervical cancer and other prevalent cancers in women. Following the group sessions, individualized follow-up consultations were provided as needed.

**Fig. 2:**
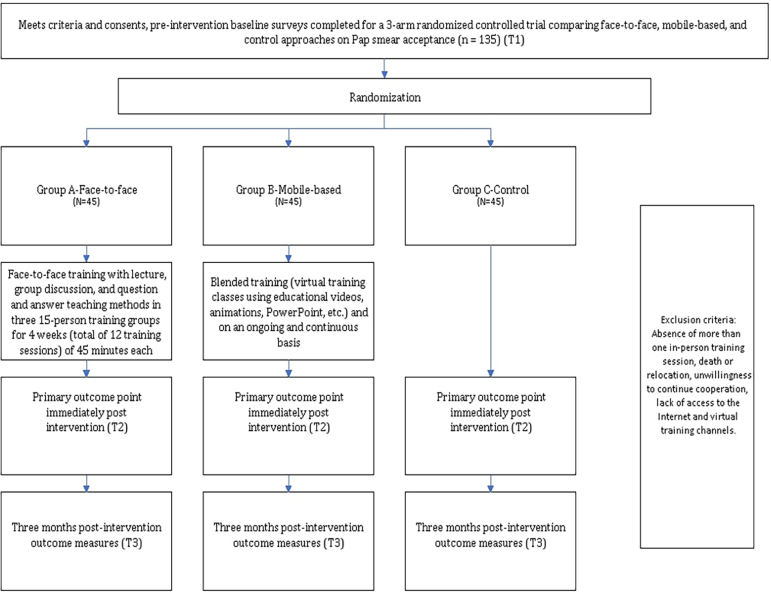
Flow diagram for a Three-Arm Randomized Controlled Trial of married, adult women Comparing Face-to-Face, Mobile-Based, and Control Approaches on Pap Smear Acceptance, Iran (2023–2024).

The survey was conducted in three stages: pre-intervention, immediate post-intervention, and follow-up three months after the intervention (Fig. 2).

### 2.5 Data collection tools

For data collection, a validated and reliable questionnaire developed by Dr. Ghafari and colleagues was used (Sharifipour et al., 2022). The reliability of the questionnaire was re-evaluated by the research team.

**Section 1 (Demographic Information):** This section included 10 questions covering age, education, marital status, medical history, economic status, contraception method, family history of disease, and occupation.

**Section 2 (Awareness about Cervical Cancer and the Pap Smear Test):** This section comprised 15 multiple-choice questions, with a scoring range of 0 to 15. Respondents selected one option per question. Correct answers were awarded 1 point, while incorrect or “I don't know” responses received 0 points. An example question is: “What disease is the Pap smear test used to diagnose?” with the options: (a) liver disease, (b) tuberculosis, (c) cervical cancer, (d) I don't know.

**Section 3 (HBM Constructs):** This section of the questionnaire evaluated the constructs of the HBM, including: Perceived Susceptibility (7 questions; scoring range: 7–35), Perceived Severity (7 questions; scoring range: 7–35), Perceived Benefits (6 questions; scoring range: 6–30), Perceived Barriers (8 questions; scoring range: 8–40), Self-Efficacy (7 questions; scoring range: 7–35), and Awareness (15 questions; scoring range: 0–15).

Scoring was based on a 5-point Likert scale: “Strongly Disagree” = 1, “Disagree” = 2, “Neutral” = 3, “Agree” = 4, and “Strongly Agree” = 5. Some items were reverse-scored. Final scores for each construct were standardized to a scale of 0 to 100 to facilitate more accurate comparisons between variables.

**Section 4 (Behavioral Intention / Behavior):** This section assessed each construct—Behavioral Intention and Behavior—using a single question, with responses recorded as either “Yes” or “No.”

### 2.6 Data analysis

Descriptive analyses were conducted to calculate the mean, standard deviation, frequency, and percentage frequency. One-way analysis of variance (ANOVA) was employed to compare age and age at marriage across the study groups, while the chi-square test was used to examine differences in education level, marital status, family history of disease, number of pregnancies, contraceptive method, occupation, and income. To address the research questions, repeated measures ANOVA (RMANOVA) was used to compare the mean scores of awareness regarding Pap smear tests, perceived risk, perceived severity, perceived benefits, perceived barriers, and perceived self-efficacy among the three groups (face-to-face, mobile-based, and control) at baseline, immediately after the intervention, and three months post-intervention. Kendall's tau was used to assess correlations across the three time points. The normality test confirmed that all variables were normally and symmetrically distributed. Analysis of covariance (ANCOVA) was employed to adjust for continuous confounding variables in the study. A two-sided *P-value* < 0.05 was set as the significance level. Data were analyzed using SPSS version 20.0 for Windows (SPSS Inc., Chicago, IL, USA).

## 3 Results

### 3.1 Descriptive results

Variables such as age, marital status, and method of contraception did not differ significantly across the groups. The only variable that showed a statistically significant difference among the groups was age at marriage (*P* < 0.05), with the mobile-based group exhibiting a higher average age at marriage compared to the other groups. These findings suggest that, overall, the study groups were more homogeneous than initially anticipated prior to the intervention (Table 1).

**Table 1:** Distribution and comparison of demographic characteristics across study groups before the educational intervention among women in Alborz, Iran (2023–2024).

**Variable**	**Control** **n (%)**	**In-Person** **n (%)**	**Mobile-Based** **n (%)**	*P-value* *
**Educational Level**				**0.43**
**Elementary/Literacy**	**7(15.6)**	**9(20)**	**5(11.1)**	
**Middle School**	**8(17.8)**	**8(17.8)**	**9(20)**	
**High School Diploma**	**22(48.9)**	**20(44.4)**	**24(53.3)**	
**University Degree**	**8(17.8)**	**8(17.8)**	**7(15.6)**	
**Marital Status**				**0.13**
**Married**	**43(95.6)**	**42(93.3)**	**39(86.7)**	
**Divorced**	**1(2.2)**	**1(2.2)**	**4(8.9)**	
**Widowed**	**1(2.2)**	**2(4.4)**	**2(4.4)**	
**Family History of Disease**				**0.08**
**Yes**	**1(2.2)**	**0**	**4(8.9)**	
**No**	**44(97.8)**	**45(100)**	**41(91.1)**	
**Number of Pregnancies**				**0.24**
**None**	**2(4.4)**	**2(4.4)**	**1(2.2)**	
**1**	**11(24.4)**	**6(13.3)**	**7(15.6)**	
**2**	**17(37.8)**	**15(33.3)**	**21(46.7)**	
**3**	**10(22.2)**	**17(37.8)**	**11(24.4)**	
**More than 3**	**5(11.1)**	**5(11.1)**	**5(11.1)**	
**Contraceptive Methods**				**0.48**
**Pills**	**5(11.1)**	**1(2.2)**	**1(2.2)**	
**IUD**^1^	**1(2.2)**	**3(6.7)**	**6(13.3)**	
**Permanent Methods**	**2(4.4)**	**5(11.1)**	**2(4.4)**	
**Injections**	**2(4.4)**	**1(2.2)**	**0**	
**Condom**	**4(8.9)**	**9(20)**	**9(20)**	
**Natural**	**24(53.3)**	**19(42.2)**	**18(40)**	
**None**	**7(15.6)**	**7(15.6)**	**9(20)**	
**Occupation**				**0.08**
**Homemaker**	**29(64.4)**	**27(60)**	**24(53.3)**	
**Employee**	**6(13.3)**	**7(15.6)**	**7(15.6)**	
**Self-employed**	**9(20)**	**11(24.4)**	**10(22.2)**	
**Other**	**1(2.2)**	**0**	**4(8.9)**	
**Monthly income (USD)**				**0.30**
**Less than140**	**19(42.2)**	**15(33.3)**	**15(33.3)**	
**140–300**	**19(42.2)**	**22(48.9)**	**23(51.1)**	
**More than 300**	**7(15.6)**	**8(17.8)**	**7(15.6)**	
	**Control** **Mean (SD)**	**In-Person** **Mean (SD)**	**Mobile-Based** **Mean (SD)**	*P-value* **
**Age (Years)**	**38.51(8.86)**	**40.16(7.63)**	**39.40(6.75)**	**0.60**
**Age at Marriage**	**18.24(2.83)**	**20.31(4.19)**	**21.80(4.48)**	**< 0.05**

* Chi-Square Test ** ANOVA Test.

1 Intrauterine Device.

### 3.2 Primary outcome

As shown in Table 2, differences in behavioral intention and behavior were evident across all evaluation time points—before, immediately after, and three months post-intervention. For behavioral intention, the mean ranks increased immediately after the intervention, with only minor changes observed at the three-month follow-up. The Kendall's W and Chi-square tests for behavioral intention indicated significant changes in rankings, with a notable increase in the difference in *P* value from pre- to post-intervention (*P* < 0.01). A similar trend was observed for behavior, where both the immediate post-intervention and three-month follow-up assessments showed an increase in rank (*P* < 0.01). In conclusion, these findings demonstrate a clear impact of the educational intervention on both behavioral intention and actual behavior, as evidenced by the increased ranks across the three time points.

**Table 2:** Changes in intention and cervical cancer screening behavior before, immediately after, and three months following an educational intervention among women in Alborz, Iran (2023–2024).

Variable / Times (Intervention)	Mean Ranks	Kendall's *P-value*
Intention		< 0.01
Before	1.81	
Immediately	2.14	
Three months	2.05	
Behavior		< 0.01
Before	1.79	
Immediately	2.06	
Three months	2.16	

### 3.3 Secondary outcomes

As shown in Table 3, significant differences were found across all psychological variables before, immediately after, and three months post-intervention. Awareness scores increased notably in both in-person and mobile-based groups, with significant within- and between-group effects (*P* < 0.01). Perceived susceptibility and severity also showed significant improvements, especially in the mobile-based group. While perceived benefits rose post-intervention, this effect was not sustained over time (*P* = 0.08). Perceived barriers decreased significantly within groups (P < 0.01), but no significant differences were observed between groups (*P* = 0.45). Perceived self-efficacy improved over time (P < 0.01), particularly in the mobile-based group, although between-group differences were not significant (*P* = 0.13). Overall, both intervention methods positively influenced psychological constructs, with mobile-based delivery demonstrating relatively stronger short- and mid-term effects.

**Table 3:** Changes in health belief model constructs before and after an educational intervention on cervical cancer screening among women in Alborz, Iran (2023–2024).

Variable/ Group	Before Intervention	Immediately After	3 Months After	*P-value* (Within)	*P-value* (Between)
Awareness				<0.01	<0.01
Control	9.13 ± 4.81	9.69 ± 4.78	10.09 ± 5.02		
In-Person	8.44 ± 4.88	16.93 ± 2.57	16.36 ± 3.39		
Mobile-Based	9.66 ± 4.19	18.61 ± 1.93	16.66 ± 3.36		
Perceived Susceptibility				<0.01	<0.01
Control	22.49 ± 5.19	22.76 ± 5.15	22.82 ± 5.28		
In-Person	22.48 ± 5.25	26.43 ± 4.19	25.18 ± 4.60		
Mobile-Based	24.27 ± 3.96	27.47 ± 3.62	25.64 ± 3.76		
Perceived Severity				<0.01	<0.01
Control	25.18 ± 4.78	25.60 ± 4.58	25.47 ± 4.44		
In-Person	26.40 ± 6.46	29.22 ± 3.80	28.18 ± 4.23		
Mobile-Based	26.24 ± 4.81	29.62 ± 4.19	28.20 ± 4.88		
Perceived Benefits				<0.01	0.08
Control	25.98 ± 4.07	26.07 ± 4.15	26.20 ± 4.10		
In-Person	25.04 ± 4.72	26.98 ± 2.86	26.13 ± 3.02		
Mobile-Based	26.51 ± 3.32	28.22 ± 2.23	27.56 ± 2.95		
Perceived Barriers				<0.01	0.45
Control	29.96 ± 7.44	30.13 ± 7.57	29.33 ± 7.38		
In-Person	27.96 ± 6.44	32.40 ± 5.47	30.42 ± 5.54		
Mobile-Based	29.87 ± 5.93	33.16 ± 4.43	31.02 ± 5.52		
Perceived Self-Efficacy				0.01	0.13
Control	26.87 ± 6.18	27.04 ± 6.19	27.11 ± 6.15		
In-Person	25.50 ± 6.46	28.09 ± 5.06	27.11 ± 5.18		
Mobile-Based	27.91 ± 4.61	29.91 ± 4.42	28.84 ± 4.70		

## 4 Discussion

This study was conducted to compare the effects of theory-based educational interventions delivered via face-to-face and mobile-based methods on Pap smear test uptake among married women.

Based on the findings of the present study, a significant increase in mean awareness scores was observed immediately and three months after the educational intervention in the control, face-to-face, and mobile-based groups. Recent studies support the effectiveness of educational interventions in enhancing women's knowledge about the Pap smear test.

Mohammad et al. (2022) demonstrated that a theory-based educational intervention combined with WhatsApp follow-up significantly improved Pap smear uptake among postnatal women in Malaysia, with a notable increase from 39.8 % to 67.9 % in the intervention group (Mohammad et al., 2022). Similarly, Romli et al. (2020) found that a health education program enhanced knowledge and attitudes toward cervical cancer and increased Pap smear uptake among female entrepreneurs in Malaysia (Romli et al., 2020). Furthermore, Drokow emphasized the importance of video materials in raising primary awareness of the Pap smear test and increasing willingness to undergo testing among Ghanaian women (Drokow et al., 2021). Additionally, a meta-analysis highlights the significance of fostering favorable attitudes and high perceived behavioral control in facilitating Pap smear uptake, suggesting that educational interventions should also address these psychological factors (Purnamasari et al., 2024). Conversely, some studies present opposing views. For example, Salehiniya et al. (2021) emphasized that various barriers, including cultural and systemic factors, limit the effectiveness of educational interventions, suggesting that knowledge alone may be insufficient to change behavior (Salehiniya et al., 2021). Similarly, Sharma et al. (2022) noted that despite theory-based interventions, minority women continue to face significant barriers to accessing Pap smear tests, indicating that additional strategies are needed to address these disparities (Sharma et al., 2022).

According to the results of the present study, there was a significant difference in the average perceived susceptibility scores immediately after the educational intervention and even three months later, compared to the control, face-to-face, and mobile intervention groups. However, the mobile group demonstrated the greatest increase in perceived susceptibility immediately following the intervention. Research indicates that educational programs based on the HBM significantly enhance women's perceived susceptibility and knowledge regarding Pap smears. For instance, one study found that women who received HBM-based education via Telegram demonstrated increased knowledge and a higher rate of Pap smear uptake three months after the intervention (Khademolhosseini et al., 2017). Recent studies support the application of mobile education approaches for promoting Pap smear testing in women. For example, a community-based controlled trial in Malaysia demonstrated that incorporating text messages into health education increased the percentage of patients who complied with Pap smear instructions from forty-eight to 79 % **(**Sharifipour et al., 2022**)**. These findings suggest that mobile-based, theory-driven educational strategies are effective in improving awareness and acceptance of Pap smear testing, highlighting the need for integrated approaches in public health initiatives. Similarly, another study demonstrated that in-person educational sessions improved women's attitudes and practices regarding Pap smears (Samami et al., 2021). However, some studies report mixed results. For example, a study conducted in rural Indonesia found that, although knowledge increased, perceived susceptibility did not change significantly following the intervention (Oktaviana and Yusuf, 2021). Additionally, a qualitative study in South Africa highlighted that, despite general awareness of cervical cancer, many women lacked specific knowledge about the Pap smear procedure, indicating a gap in effective education (Gwavu et al., 2023). Furthermore, some studies do not support the effectiveness of mobile-based educational interventions for Pap smear testing. For instance, a study in Texas found that a text messaging intervention did not significantly increase Pap smear uptake compared to usual care, although it showed marginal benefits for vulnerable populations, such as low-income women (Bhardwaj et al., 2023b). Similarly, a study in India reported only a 5 % increase in Pap smear uptake following an mHealth intervention, indicating limited effectiveness in improving screening rates among women from low socioeconomic backgrounds (Hombaiah et al., 2022).Additionally, a systematic review highlighted that, while mobile technologies can raise awareness, their impact on actual screening uptake remains inconclusive, suggesting a need for further research (Mariño et al., 2023). Collectively, these findings suggest that although mobile-based interventions may enhance knowledge and awareness, their effectiveness in increasing actual Pap smear testing rates is not consistently supported.

The present findings indicated that while changes in perceived barriers were significant at the within-group level, the differences between groups did not reach statistical significance. Research on Asian women revealed that various sociodemographic factors, including awareness and cultural beliefs, significantly impact cervical cancer screening, emphasizing the need for targeted educational programs (Salehiniya et al., 2021). A study among highly educated Indian women found a favorable attitude toward screening, yet only a small percentage had undergone Pap tests, indicating a gap between perception and practice (Agarwal et al., 2022). A study in Saudi Arabia reported that despite high awareness of cervical cancer, only 2 % of women had undergone Pap smears, suggesting significant barriers related to perceived susceptibility and knowledge (Ibrahim et al., 2022). Research in Nigeria identified fear and lack of knowledge as major barriers to screening, contrasting with findings that suggest education improves screening rates (Ubah et al., 2022). Additionally, a qualitative study in the U.S. highlighted that knowledge gaps persist even among women with positive perceptions of healthcare communication, indicating that perceived barriers can vary widely (Kulkarni et al., 2023).

The present study showed that the mean perceived self-efficacy score significantly improved over time; however, the difference between the face-to-face and mobile-based groups was not statistically significant. Notably, the mobile-based group demonstrated the greatest increase in self-efficacy. Concurrent studies also suggest that mobile-based interventions can effectively increase Pap smear uptake. For example, a study in Malaysia found that mobile technology enhanced Pap smear uptake among women during the postpartum period and improved their self-efficacy and knowledge (Mohammad et al., 2022). Moreover, a study in Nigeria highlighted the role of mobile health approaches in improving adherence to Pap smear screening (Okunade et al., 2020). In contrast, a study conducted in Texas found that text message interventions did not influence Pap smear uptake compared to standard care procedures (Bhardwaj et al., 2023a). Overall, it can be concluded that mobile interventions have the potential to enhance self-efficacy and screening participation, although their effectiveness may depend on specific contexts and the educational methods employed. Further research is needed to explore the effective adaptation of these interventions for diverse populations.

## 5 Strengths and limitations

The strengths of this study include its randomized controlled design, the use of the HBM to guide educational content, and the direct comparison of face-to-face and mobile-based interventions, offering valuable insights into their feasibility in low-resource settings. Limitations included participant time constraints, financial barriers, and a relatively short three-month follow-up period, which may not fully capture long-term behavior change. However, since Pap smear screening is a one-time preventive action, its occurrence within this period still reflects the intervention's effectiveness.

### 5.1 Suggestions for future research

Future studies should include longer follow-up periods to assess sustained behavior change and repeat Pap smear testing. Qualitative research is also needed to explore barriers to screening, and evaluating the cost-effectiveness and scalability of mobile interventions could inform health policy.

### 5.2 Transferability of findings to diverse populations

The findings may be transferable to socio-cultural and healthcare contexts similar to those studied, particularly in low- and middle-income countries. Theory-based and mobile interventions are adaptable; however, they must be tailored to local needs, literacy levels, and infrastructure to ensure effectiveness.

## 6 Conclusion

The results of this study underscore the importance of structured, theory-based educational interventions—particularly mobile-based strategies—as effective tools for improving Pap smear acceptance among women. Given their scalability, cost-effectiveness, and accessibility, mobile-based educational tools can serve as a vital complement to face-to-face education, particularly in settings with limited healthcare infrastructure.

## Financial support and sponsorship

This study did not receive financial support from any organization.

## Artificial intelligence disclosure

Artificial intelligence (AI) was utilized solely for the purpose of improving the grammar, phrasing, and clarity of the English language in the preparation of this manuscript. The AI tool used was ChatGPT (OpenAI, San Francisco, CA, USA). No part of the content, data analysis, or scientific interpretation was generated by AI. The authors take full responsibility for the integrity and accuracy of the manuscript.

## Credit authors statement

AB and ASM designed the study. AB and ASM wrote the first draft. MR and ASM conducted the analyses. All authors contributed to writing, revising, and approved the final manuscript.

## CRediT authorship contribution statement

**Azar Bahrami:** Writing – review & editing, Writing – original draft, Methodology. **Mitra Rahimzadeh:** Software, Methodology, Formal analysis. **Ali Safari-Moradabadi:** Writing – review & editing, Writing – original draft, Supervision, Methodology, Conceptualization.

## Consent for publication

Not applicable.

## Ethical considerations

In this study, all material and intellectual rights of the participants were respected, and the confidentiality of their information was ensured, with results published anonymously. Informed written consent was obtained from all participants prior to the commencement of the study, and participants retained the right to withdraw at any time.

## Declaration of competing interest

The authors declare that they have no known competing financial interests or personal relationships that could have appeared to influence the work reported in this paper.

## Data Availability

Data will be made available on request.

## References

[bb0005] Agarwal M., Sinha S., Singh G., Singh S., Ahmad S., Sinha S., Singh G. (2022). Attitude and perceived barriers among highly educated women towards cervical cancer screening by pap smear: an online survey. Cureus.

[bb0010] Ahadinezhad B., Maleki A., Amerzadeh M., Mohtashamzadeh B., Khosravizadeh O. (2024). What rate of Iranian women perform pap smear test? Results from a meta-analysis. Prev. Med..

[bb0015] AziziKia H., Didar H., Teymourzadeh A., Nakhostin-Ansari A., Doudaran P.J., Ferasatifar B., Hoveidaei A., Roshandel G. (2023). Uterine and cervical cancer in Iran: an epidemiologic analysis of the Iranian National Population-Based Cancer Registry. Arch. Iran. Med..

[bb0020] Balou H.A., Joukar F., Yeganeh S., Hassanipour S., Naghipour M., Baghaee M., Sadeghi G., Hayadokht G., Nikbakht H.A., Mansour-Ghanaei F. (2022). Epidemiological study of different types of cancers among adults Persian Guilan cohort study [applicable]. J. Guilan Univ. Med. Sci..

[bb0025] Bhardwaj N., Herndon A.T., Kuo Y.-F., Porterfield L.R. (2023). Text messaging intervention for pap smear uptake: a single-institution study. Mhealth.

[bb0030] Drokow E.K., Effah C.Y., Agboyibor C., Sasu E., Amponsem-Boateng C., Akpabla G.S., Ahmed H.A.W., Sun K. (2021). The impact of video-based educational interventions on cervical cancer, pap smear and HPV vaccines. Front. Public Health.

[bb0035] Eftekharzadeh S., Ebrahimi N., Samaei M., Mohebi F., Mohajer B., Sheidaei A., Gohari K., Moghaddam S.S., Ahmadi N., Fateh S.M. (2020). National and subnational trends of incidence and mortality of female genital cancers in Iran; 1990–2016. Arch. Iran. Med..

[bb0040] Ghoreishi M.S., Peyman N., Abusalehi A. (2019). The effect of educational intervention based on the health belief model on using vitamin D supplements among female high school students in Mashhad. Health Dev. J..

[bb0045] Gwavu Z., Murray D., Okafor U.B. (2023). Perception of women’s knowledge of and attitudes towards cervical cancer and Papanicolaou smear screenings: a qualitative study in South Africa. Healthcare.

[bb0050] Hatefnia E., Ghazivakili Z. (2015).

[bb0055] Hombaiah C. Madhu B. Gopi A. Narayana Murthy M. Effects of mobile health (mHealth) application on cervical cancer prevention knowledge and screening among women social support groups with low-socioeconomic status in Mysuru city, Southern India PLoS One 17 9 e0273070. 10.1371/journal.pone.0273070 PMC9436151 36048892

[bb0060] Hoshmndi K., Hoveizi E., Gooraninejad S., Tabandeh M. (2020). Comparison of the apoptotic effects of TPSF small molecule and hydroalcoholic extract of *Ziziphus spina-christi* on cervical cancer cells. Feyz J. Kashan Univ. Med. Sci..

[bb0065] Ibrahim H.A., Nahari M.H., Al-Thubaity D.D., Alshahrani M.A., Elgzar W.T., El Sayed H.A., Sayed S.H. (2022). Saudi women health beliefs and associated factors regarding cervical cancer prevention at Najran city: a theory-based study. Afr. J. Reprod. Health.

[bb0070] Khademolhosseini F., Noroozi A., Tahmasebi R. (2017). The effect of health belief model-based education through telegram instant messaging services on pap smear performance. Asian Pac. J. Cancer Prev..

[bb0075] Kulkarni A., Glynn S., Gamble C.R., Shen M.J., Cantillo E., Frey M.K., Holcomb K.M., Safford M.M., Chapman-Davis E. (2023). Understanding perceived barriers to colposcopy follow-up among underserved women at an urban teaching hospital: a qualitative study. J. Low. Genit. Tract Dis..

[bb0080] Mariño J.M., Nunes L.M.P., Ali Y.C.M.M., Tonhi L.d.C., Salvetti M.d.G. (2023). Educational interventions for cervical cancer prevention: a scoping review. Rev. Bras. Enferm..

[bb0085] Massobrio R., Bianco L., Campigotto B., Attianese D., Maisto E., Pascotto M., Ruo Redda M.G., Ferrero A. (2024). New frontiers in locally advanced cervical cancer treatment. J. Clin. Med..

[bb0090] Mohammad Z., Ahmad N., Baharom A. (2022). The effects of theory-based educational intervention and WhatsApp follow-up on papanicolaou smear uptake among postnatal women in Malaysia: randomized controlled trial. JMIR Mhealth Uhealth.

[bb0095] Mokhtari Nouri J., Jalali M., Parandeh A., Javadi M. (2019). The effectiveness of education based on health belief model on promoting preventive behaviors of mothers from girls' early puberty [quantitative-research]. J. Nurs. Educ..

[bb0100] Nazari S., Keshavarz Z., Afrakhte M., Riazi H. (2019). Barriers to cervical cancer screening in HIV positive women: a systematic review of recent studies in the world. Iran. J. Epidemiol..

[bb0105] Nejati A., Mirteimouri M., Nikdoust S., Nikdoust M., Alizadeh F. (2020). Human papillomavirus (HPV) test and pap smear in screening for cervical cancer: a systematic review on systematic review study. Iran. J. Obstet. Gynecol. Infertil..

[bb0110] Oktaviana D., Yusuf R.A. (2021). The effect of health education based on the health belief model about pap smear test on women in rural district Indonesia. Medico-Legal Update..

[bb0115] Okunade K.S., Salako O., Adejimi A.A., Akinsola O.J., Fatiregun O., Adenekan M.A., Moses O.E., Ebenso B., Allsop M.J., Anorlu R.I. (2020). Impact of mobile technologies on Cervical cancer screening practices in Lagos, Nigeria (mHealth-Cervix): protocol for a randomised controlled trial. F1000Res.

[bb0120] Purnamasari E., Demartoto A., Budihastuti U.R. (2024). Application of theory of planned behavior on factors associated with pap smear uptake: a meta-analysis. J Health Promot Behav.

[bb0125] Romli R., Saddki N., Mokhtar N. (2020). Effectiveness of a health education program to improve knowledge and attitude towards cervical cancer and Pap smear: a controlled community trial in Malaysia. Asian Pac. J. Cancer Prev..

[bb0130] Rostami S., Nahvijou A. (2022). Gynecologic cancers estimates in the IR Iran, 2012–2040. Basic Clin. Cancer Res..

[bb0135] Salehiniya H., Momenimovahed S., Allahqoli L., Momenimovahed Z., Alkatout I. (2021). Factors related to cervical cancer screening among Asian women. Eur. Rev. Med. Pharmacol. Sci..

[bb0140] Samami E., Seyedi-Andi S.J., Bayat B., Shojaeizadeh D., Tori N.A. (2021). The effect of educational intervention based on the health belief model on knowledge, attitude, and function of women about pap smear test at Iranian health centers: a randomized controlled clinical trial. J. Educ. Health Promot..

[bb0145] Sharifi M., Mohammadi Z., Makvandi Z., Rostami P., Moradi A. (2018). Assessment of cervical cancer screening and its barriers in 18-50 year old women referring to Asad Abad comprehensive health centers. Pajouhan Sci. J..

[bb0150] Sharifipour Z., Rakhshanderou S., Mehrabi Y., Safari-Moradabadi A., Ghaffari M. (2022). Women’s decision to adopt or not adopt cervical cancer screening: application of precaution adoption process model as the theoretical framework. J. Educ. Health Promot..

[bb0155] Sharma M., Batra K., Johansen C., Raich S. (2022). Explaining correlates of cervical cancer screening among minority women in the United States. Pharmacy.

[bb0160] Shokouhi F., Amiripour A., torabi z., Rabiei L. (2021). Application of health belief model on nutritional behavior change in women with type 2 diabetes in Shahrekord. Sci. J. Nurs. Midwifery Paramed. Fac..

[bb0165] Ubah C., Nwaneri A.C., Anarado A.N., Iheanacho P.N., Odikpo I. (2022). Perceived barriers to cervical cancer screening uptake among women of an urban community in South-Eastern Nigeria. Asian Pac. J. Cancer Prev..

[bb0170] Zareai M. (2014). Women's knowledge and practice of pap smear test and barriers to performing it in Jahrom [research]. Pars Jahrom Univ. Med. Sci..

